# Structure and Electrical Behavior of Hafnium-Praseodymium Oxide Thin Films Grown by Atomic Layer Deposition

**DOI:** 10.3390/ma15030877

**Published:** 2022-01-24

**Authors:** Kaupo Kukli, Lauri Aarik, Guillermo Vinuesa, Salvador Dueñas, Helena Castán, Héctor García, Aarne Kasikov, Peeter Ritslaid, Helle-Mai Piirsoo, Jaan Aarik

**Affiliations:** 1 Institute of Physics, University of Tartu, W. Ostwaldi 1, 50411 Tartu, Estonia; lauri.aarik@ut.ee (L.A.); aarne.kasikov@ut.ee (A.K.); peeter.ritslaid@ut.ee (P.R.); helle-mai.piirsoo@ut.ee (H.-M.P.); jaan.aarik@ut.ee (J.A.); 2 Department of Electronics, University of Valladolid, Paseo Belén 15, 47011 Valladolid, Spain; guillermo.vinuesa@alumnos.uva.es (G.V.); sduenas@ele.uva.es (S.D.); helcas@tel.uva.es (H.C.); hecgar@tel.uva.es (H.G.)

**Keywords:** hafnium oxide, praseodymium oxide, atomic layer deposition, crystal structure, dielectric properties, resistive switching

## Abstract

Crystal structure and electrical properties of hafnium-praseodymium oxide thin films grown by atomic layer deposition on ruthenium substrate electrodes were characterized and compared with those of undoped HfO_2_ films. The HfO_2_ reference films crystallized in the stable monoclinic phase of HfO_2_. Mixing HfO_2_ and PrO_x_ resulted in the growth of nanocrystalline metastable tetragonal HfO_2_. The highest relative permittivities reaching 37–40 were measured for the films with tetragonal structures that were grown using HfO_2_:PrO_x_ cycle ratio of 5:1 and possessed Pr/(Pr + Hf) atomic ratios of 0.09–0.10. All the HfO_2_:PrO_x_ films exhibited resistive switching behavior. Lower commutation voltages and current values, promising in terms of reduced power consumption, were achieved for the films grown with HfO_2_:PrO_x_ cycle ratios of 3:1 and 2:1 and showing Pr/(Pr + Hf) atomic ratios of 0.16–0.23. Differently from the undoped HfO_2_ films, the Pr-doped films showed low variability of resistance state currents and stable endurance behavior, extending over 10^4^ switching cycles.

## 1. Introduction

HfO_2_, as a high-permittivity metal oxide, has attracted marked attention as a functional component of different nanoelectronic devices. HfO_2_ films have been studied and exploited as gate dielectric layers in both planar [1] and three-dimensional (3D) Fin-type [2] field-effect transistors. In order to modify the phase composition and enhance the functionality of the films, HfO_2_ has been doped with foreign metals or metal oxides. For example, Al-doped phase-stabilized HfO_2_ has been investigated as a dielectric for volatile dynamic random access memories [3]. In addition, stabilization of orthorhombic phase of HfO_2_ by doping with aluminum [4,5] or praseodymium [6] has been reported to cause the appearance of ferroelectric behavior, opening up routes to HfO_2_-based nonvolatile memories.

Regarding other potential applications of HfO_2_, resistive switching (RS) memory devices [7,8,9,10,11,12,13,14,15,16,17] are of significant importance. RS media based on metal oxides have been of interest for materials scientists and engineers over several decades, dating back to the beginning of 1960-ies [7]. The diversity in the choice of the materials suited to this application is still large, as one can decide on the basis of several recent reviews [7,8,9,10]. Amongst the metal oxide thin films under investigation, different compounds, such as TiO_2_, Al_2_O_3_, Ta_2_O_5_, ZrO_2_, or HfO_2_, can be considered.

HfO_2_ thin films have been examined as RS media in several studies [12,13,14,15,16,17]. At the same time, to improve the RS performance, the effect of doping has also been investigated. For instance, aluminum has been used as a dopant in RS HfO_2_ films [18,19]. In a long list of other dopants used in oxide films, praseodymium (Pr) is the one that has been applied to improve RS properties of CeO_2_ [20] and ZnO [21]. Moreover, Pr has found application as a component of a rather complex compound, Pr_0.7_Ca_0.3_MnO_3_, examined as RS media in a number of studies [22,23,24,25,26,27]. In the case of HfO_2_, Pr has mainly attracted attention as a dopant allowing stabilization of metastable phases with ferroelectric properties and high permittivity values [6]. These results led us to a conclusion that the further investigation of Pr-doped HfO_2_ as an electronic material could be of significant interest, especially because the Pr impurities might also influence the RS performance of HfO_2_.

Among the methods that can be used for the deposition of HfO_2_ thin films, atomic layer deposition (ALD) is the one that allows conformal coating of substrates with complex shapes [3]. For this reason, the method is of particular importance in the production of electronic devices with 3D integration [2,3]. The HfO_2_ thin films have been grown by ALD from different hafnium precursors [4,5,13,14,15,18,19,28] of which HfCl_4_ is a carbon-free metal precursor allowing self-limited ALD-type growth in an extremely wide temperature range extending from 225 to 940 °C [28]. H_2_O has been the most common oxygen precursor applied in ALD of HfO_2_. However, ozone (O_3_) as a hydrogen-free oxygen precursor has also been exploited for ALD together with HfCl_4_ [29,30,31,32]. As HfCl_4_ and O_3_ had advantages in the deposition of films with low contamination levels, the ALD process based on these precursors was also employed in the present work.

Praseodymium oxide (PrO_x_) thin films have been grown using chemical vapor deposition [33] as well as ALD [34] routes based on Pr(thd)_3_ (thd = 2,2,6,6-tetramethyl-3,5-heptanedione). The paper by Hansen et al. [34] implies that H_2_O does not serve as an appropriate co-reactant together with Pr(thd)_3_ because this precursor combination does not yield films with well-developed crystallinity and sufficient thickness uniformity. Another study on crystallization in lanthanide oxides [35] has indicated that PrO_x_ films can be grown at 300 °C from Pr(thd)_3_ and O_3_. However, the films tend to be of multiphase composition, containing Pr_6_O_11_ and cubic PrO_2_ phases due to the ability of this lanthanide to form Pr^3+^ and Pr^4+^ oxides and the latter’s mixed phases.

Mixed PrO_x_ and hafnium oxide films have been grown by ALD using Pr(thd)_3_, HfCl_4,_ and O_3_ as the precursors [36]. In these experiments, the effects of deposition process parameters on the growth rate, crystal structure, phase composition, and optical properties (refractive index, optical bandgap, and photoluminescence efficiency) of the films were characterized [36]. The results revealed that the main phases observed in the praseodymium oxide films were PrO_2_ formed at 225–250 °C, Pr_6_O_11_ formed at 275 °C, and Pr_7_O_12_ and Pr_6_O_11_ formed at 300–325 °C. The studies on ALD of HfO_2_ from HfCl_4_ and O_3_ [32] have revealed that the monoclinic phase is predominantly obtained in the thicker (>40 nm) films at the substrate temperatures ranging from 225 to 600 °C. However, in the thinner films, the metastable cubic, tetragonal, or orthorhombic phase has also been observed [32]. Pr-doping stimulated the growth of the metastable phases while at sufficiently high Pr concentrations; only the tetragonal *t*′-form has been obtained in the films [36].

In nanoelectronics, ruthenium (Ru), as a noble metal with a high work function, has been of interest and studied as an electrode material of dynamic random access memory capacitors [37,38,39,40] with high permittivity dielectrics and very high capacitance density values [37]. Ru has also been applied as an electrode material of RS stacks [41,42,43]. Ru electrodes of RRAM devices have been directly contacted to Nb_2_O_5_ [44], tantalum oxide [43,45], or HfO_2_ films [41]. Depending on the processes investigated, the Ru electrodes [43] and/or switching metal oxide layers [42,46] may have been grown by ALD.

Although RS can be based on several underlying mechanisms, the switching phenomena in the metal–insulator–metal stacks with metal oxide dielectrics and electrochemically inactive metal electrodes are usually governed by the valence change mechanism (VCM) [46,47]. In this type of RS media, conductive filaments are formed by oxygen vacancies and grow in the dielectric until they connect metal electrodes. To create these filaments for the first time, it is usually necessary to perform an electroforming process applying voltage values that are higher than those needed later for RS [48,49]. Thereafter, using voltage with inverted polarity, the filament can be partially disrupted (RESET event). To form the conductive filament again (SET event), a voltage with the same polarity as in the electroforming process, but with a much lower value, can be applied [50]. VCM has been suggested to be the main RS mechanism in the structures with HfO_2_ dielectric and with inert metal electrodes [51,52]. Recently, the conductive filamentary model has been described in an RS medium consisting of alloyed HfTiO_x_ films in silicon-based contact hole structures where the oxide was grown by ALD from tetrakisethylmethyl(amino) hafnium, titanium tetraisopropoxide, and water [53]. The VCM of RS has also been observed in the case of other oxides. For instance, there is experimental evidence of this filamentary conduction in Ta_2_O_5_-based films [54]. Therefore, the same RS mechanism was expected to appear in Pr-doped HfO_2_ as well.

The present work was performed to investigate the effect of Pr-doping on the electrical properties of HfO_2_, in particular, on the permittivity and RS performance. High permittivity together with high bandgap values is important in applications where HfO_2_ is used as a high-permittivity gate or capacitor dielectric. Earlier studies have revealed that Pr doping of HfO_2_ causes an increase in the bandgap values and stabilization of a metastable tetragonal phase [36] that was expected to lead to the permittivity increase. A goal of this work was to obtain experimental data on the permittivity of Pr-stabilized metastable HfO_2_. Another goal of this work was to investigate if the stabilization of the metastable phase and increase in the bandgap energy influence the RS performance, particularly the low to high resistance state ratio during RS and the stability of that ratio in terms of the endurance of such samples on ruthenium electrodes.

## 2. Materials and Methods

The Pr-doped HfO_2_ films studied in this work were deposited in an in-house built hot-wall flow-type ALD reactor [55] at 325 °C using HfCl_4_ (99.9 %, Aldrich Chemicals Co. St. Louis, MO, USA) and O_3_ as the precursors for deposition of HfO_2_ [32], and Pr(thd)_3_ (Volatec Oy, Porvoo, Finland) and O_3_ for adding PrO_x_ to the film material [36]. Nitrogen (99.999%, AS Linde Gas, Tallinn, Estonia) was the carrier as well as purging gas in these experiments. The deposition process parameters were similar to those used earlier [36]. During the deposition, the net gas pressure in the reactor was kept at 200–220 Pa. O_3_ was produced from O_2_ (99.999% purity, AS Linde Gas, Tallinn, Estonia) using a BMT 802N ozone generator (BMT Messtechnik, Stahnsdorf, Germany). The O_3_ concentration, measured by a BMT 964 ozone analyzer (BMT Messtechnik, Stahnsdorf, Germany) in the O_3_/O_2_ mixture at the outlet of the O_3_ generator, was 240–260 g/m^3^. The partial pressure of the O_3_/O_2_ mixture was set at 22 Pa in the reaction chamber during the oxygen precursor pulses.

The Ru bottom electrodes were deposited on Si(100) substrates at room temperature by DC-magnetron sputtering in Ar (99.999%) environment using a Ru (99.95%) planar target of 25 mm in diameter_._ The pressure during the sputtering process was 3 × 10^−3^ mbar, the DC-power was 2 W and the distance between target and substrate was 60 mm.

For the deposition of Pr-doped HfO_2_, supercycles—including one cycle of Pr(thd)_3_-O_3_ per 2–5 cycles of HfCl_4_-O_3_—were repeated 25–50 times to obtain 19–50-nanometer thick Pr-doped films studied in this work. Each ALD cycle used for deposition of HfO_2_ included a HfCl_4_ pulse, purge, O_3_ pulse, and another purge with durations of 5, 2, 5, and 5 s, respectively, while those used for deposition of PrO_x_ included a Pr(thd)_3_ pulse, purge, O_3_ pulse, and purge with durations of 5, 2, 5, and 5 s, respectively. No post-deposition annealing was applied for samples studied in this work.

The crystalline phases formed in the films were determined by the grazing incidence XRD (GIXRD) method using an X-ray diffractometer SmartLab (Rigaku, Tokyo, Japan) and Cu Kα radiation. The incidence angle chosen for the GIXRD measurements was 0.42°, corresponding to the scattering depth of 20 nm in cubic PrO_2_ and 11 nm in monoclinic HfO_2_ [36]. The diffractometer was also employed for X-ray reflection (XRR) measurements to determine the film thickness and surface roughness values. Additionally, the film thicknesses were measured with a GES5E spectroscopic ellipsometer (Semilab Sopra, Budapest, Hungary). Combining these two methods, the thicknesses of thinner (19–28 nm) films were determined with an accuracy better than ±2 nm while those of thicker (50–65 nm) films were obtained with an accuracy of ±3 nm. The mass thicknesses and elemental compositions of the films were determined with X-ray fluorescence (XRF) analyzer ZSX400 (Rigaku, Tokyo, Japan). Scanning electron microscope (SEM) Helios NanoLab 600 (FEI Company, Hillsboro, OR, USA) was used in a high-resolution mode (at an acceleration voltage of 10 kV and electron beam current of 86 pA) to characterize the surface microstructure.

For the electrical measurements, titanium top electrodes with circular geometry and thicknesses of 50 nm were electron-beam evaporated on the dielectric through a shadow mask at 230 °C. The Ti electrodes used in the measurements had areas of 0.002 and 0.052 mm^2^. Electrical measurements were carried out in a probe station using a Keithley 4200-SCS semiconductor analyzer (Keysight Technologies, Cleveland, OH, USA). In the DC measurements, the bias voltage was applied to the top electrode while the bottom electrode remained grounded. To initiate RS, every sample required an electroforming procedure that was carried out as a voltage sweep with positive bias using a current compliance set at 1 μA to avoid irreversible breakdown of devices. In general, the electroforming took place between 7 and 13 V. The current–voltage (I-V) curves were obtained by applying positive and negative voltage sweeps to switch between the resistance states (bipolar RS), while the memory maps [56,57] were measured by reading the current value at 0.1 V after applying every increasing (or decreasing) voltage value used in the I-V envelope curves. To carry out the endurance measurements, a high number of relatively fast RS cycles were recorded. Each cycle was defined by a voltage pulse sequence of V_set_, 0.1 V, –V_reset_, and 0.1 V with switching pulse time durations of 0.1 s for V_set_ as well as for V_reset_. Capacitance frequency measurements were carried out by applying a 30-millivolt signal without a DC bias in the 10 kHz–1 MHz frequency range.

## 3. Results and Discussion

### 3.1. Composition and Structure

**Figure 1:**
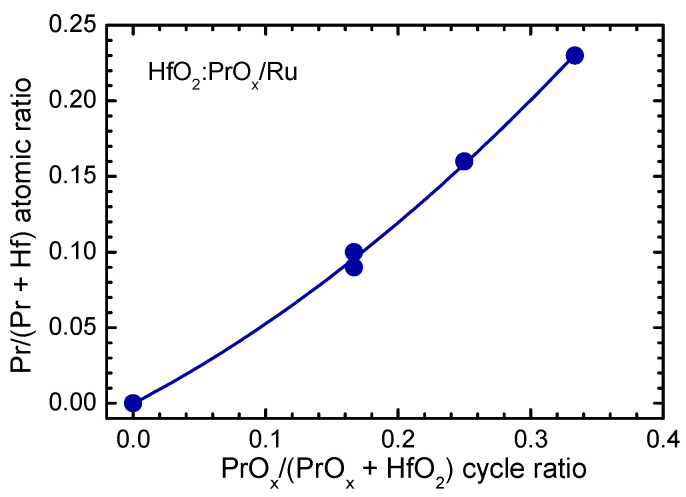
Pr/(Hf + Pr) atomic ratio as a function of PrO_x_/(HfO_2_ + PrO_x_) cycle ratio, used for ALD of HfO_2_:PrO_x_ films.

The Pr/(Hf + Pr) atomic ratio in the HfO_2_:PrO_x_ films was appreciably correlated with the ratio of HfO_2_ and Pr_2_O_3_ deposition cycles (Figure 1) and equaled to 0.23, 0.16, and 0.10 in the 28-, 23-, and 19-nanometer thick films, grown using the HfO_2_:PrO_x_ cycle ratios of 2:1, 3:1, and 5:1, respectively. In the 50-nanometer thick film grown using the HfO_2_:PrO_x_ cycle ratio of 5:1, the Pr/(Hf + Pr) atomic ratio was 0.09.

**Figure 2:**
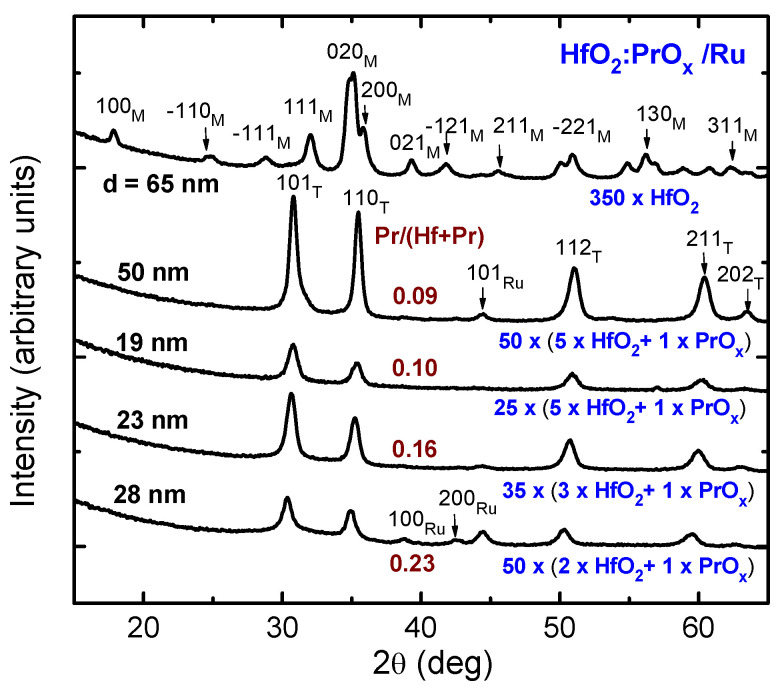
Grazing incidence diffraction patterns from HfO_2_:PrO_x_ films deposited on Ru. The growth cycle sequences for HfO_2_ and PrO_x_, and Miller indexes attributed to either tetragonal (T) or monoclinic (M) phases of HfO_2_ are shown at the diffraction patterns. Reflections from substrate Ru layers are denoted by corresponding labels. The film thicknesses (d) and compositions (Pr/(Hf + Pr) atomic ratios) are also indicated by labels.

As expected, the Pr content influenced the crystal structure of the HfO_2_:PrO_x_ films. As can be seen in Figure 2, the 65-nanometer thick HfO_2_ reference film, i.e., the one which did not contain PrO_x_, was unambiguously crystallized in the form of the stable monoclinic polymorph. Furthermore, the expected and also obvious effect of the doping was the stabilization of a metastable polymorph. It should be mentioned, however, that the distinction between metastable cubic, tetragonal, and even orthorhombic phases of HfO_2_ was quite complicated, considering the small crystallite sizes accompanied by peak broadening and possible effects of doping and substrate structure, causing shifts in the peak positions. The corresponding reflections, peaking at 30.3, 35.5, 50.3, 60.5, and 63.0 degrees (Figure 2), were attributable to the 101, 110, 112, 211, and 202 reflections of tetragonal HfO_2_ (PDF card 01-078-5756). At the same time, the reflections might also be assigned as 111, 002, 022, 113, and 222 reflections of the cubic HfO_2_ (PDF card 96-900-9017), or as 111, 020, 022, 113, and 311 reflections of the orthorhombic HfO_2_ (PDF card 00-021-0904). However, it is worth mentioning that the refinement of similar diffraction patterns of Pr-doped HfO_2_ thin films grown by ALD on silicon substrates to thicknesses of 140–328 nm revealed the formation of tetragonal *t*′ form rather than other phases of HfO_2_ in the films with Pr/(Pr + Hf) atomic ratios ≥ 0.095 [36]. The tetragonal *t*′ form [58] is characterized by the relatively small tetragonality of its lattice (with lattice parameter ratios *c*/*a* ranging from 1.004 to 1.012 for the pseudo-fluorite unit cell), and therefore is an intermediate structure form between the cubic and tetragonal HfO_2_. Nevertheless, it is also possible that the structure of the relatively thin films deposited on Ru in the present work had not been fully formed and, for this reason, was of a multiphase nature. In particular, low GIXRD reflection intensities of the thinner films grown with a HfO_2_:PrO_x_ cycle ratio of 5:1 indicated the presence of amorphous and/or nanocrystalline phases with poorly developed structure in the films.

Although no direct correlation between the resistance state values and dielectric thickness had been observed in the VCM type RS media, based on HfO_2_ [59] and other media [60], the dependence of the permittivity and RS performance on the structure development related to the dielectric thickness (Figure 2) could not be neglected. However, the results of electrical measurements presented below were dominantly influenced by the chemical and phase composition rather than by the thickness of the dielectric. For this reason, the further downscaling of the thickness was not studied in the present work.

**Figure 3:**
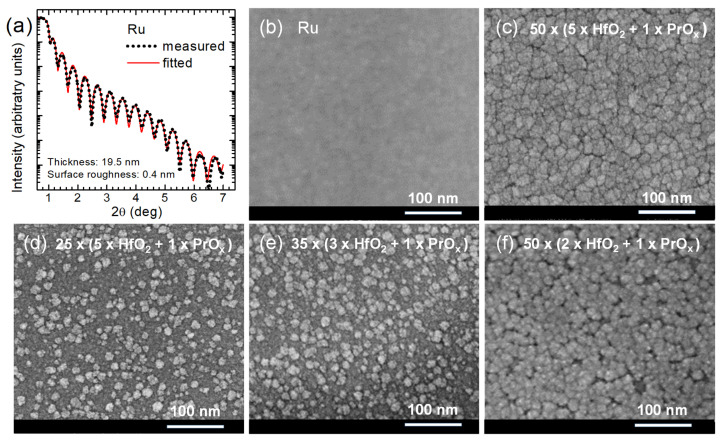
(**a**) XRR pattern on bare Ru bottom electrode, and (**b**–**f**) SEM images of (**b**) bare Ru bottom electrode, and (**c**–**f**) surfaces of HfO_2_:PrO_x_ films deposited using cycle sequences indicated by labels.

The fitting of XRR data, measured for a 19.5-nanometer-thick Ru film used as a bottom electrode (Figure 3a), yielded a surface roughness value as low as 0.4 ± 0.2 nm. Thereby, the density of Ru was found to be 12.5 g/cm^3^, that is, close to the bulk value. The SEM studies confirmed the almost featureless and artifact-free nature of Ru electrode films (Figure 3b), allowing one to rely on smooth contact interfaces between the electrode and the RS medium. This was an important result because the SET events of the RS processes took place at positive voltages applied to the top electrodes. In such a case, the smoothness of the bottom electrode was assumed to reduce the parasitic leakage currents related to the electronic conduction and allowed us to expect reliable and reproducible electrical performance, uniform and stable over the electrode matrix formed on a sample used for electrical studies.

The thicker dielectric film grown using HfO_2_:PrO_x_ cycle ratio of 5:1 evidently consisted of densely packed grains (Figure 3c). A surface roughness of 1.9 nm, obtained from XRR measurements of this film, was notably higher than that of the Ru surface. For comparison, the surface roughness of a HfO_2_ film of similar thickness was 1.7 nm. Smaller but distinctive grain-like features were observed by SEM on the surfaces of 2–3 times thinner oxide films (Figure 3d–f). The surface roughness values of these films grown using the HfO_2_:PrO_x_ cycle ratios of 5:1, 3:1, and 2:1 were 0.6, 1.4, and 1.2 nm, respectively. It is worth noting that the SEM images as well as surface roughness values were consistent with the degrees of crystallization characterized by GIXRD (Figure 2).

### 3.2. Dielectric Properties

**Figure 4:**
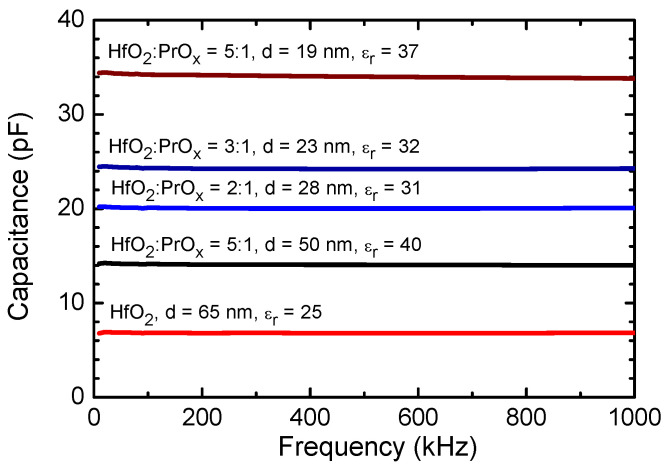
Capacitance dispersion curves measured from HfO_2_:PrO_x_ films deposited. HfO_2_:PrO_x_ cycle ratios and film thicknesses are indicated by labels. Indicated are also the average relative permittivity values calculated at 100 kHz using the common parallel plate capacitor stack formula. The area of the capacitor electrodes used was 0.052 mm^2^. The uncertainty of the measurements did not exceed 10%.

Figure 4 depicts the results of capacitance frequency measurements carried out on HfO_2_:PrO_x_ films deposited on common Ru bottom electrodes and supplied with top electrodes arranged in a matrix. The capacitance did not show marked dispersion in a frequency range of 10–1000 kHz (Figure 4). This result could be considered as a plausible indication of the insignificant role of the relatively free space charge. Expectedly and most clearly, the capacitance correlated to the film thickness. However, the relative permittivity that depended on the phase composition of the HfO_2_:PrO_x_ films (Figure 2) also influenced the capacitance values.

The relative permittivity values were calculated from the mean capacitance values measured at 100 kHz. The highest permittivities, 37 and 40, were achieved from the calculations for the films deposited using a HfO_2_:PrO_x_ cycle ratio of 5:1 to the thicknesses of 19 and 50 nm, respectively. The GIXRD data (Figure 2) and results of the more complex analysis performed earlier [36] indicated that both films contained tetragonal phase (*t*′ form), with the most intense reflections of 101 and 110, apparent in their diffraction patterns. The 23 and 28 nm thick films that were grown using HfO_2_:PrO_x_ cycle ratios of 3:1 and 2:1, and crystallized in the same metastable polymorph, possessed relative permittivities of 32 and 31, respectively. The lowest permittivity value that was equal to 25 was measured for the undoped HfO_2_ film, which was grown to the thickness of 65 nm, and contained monoclinic HfO_2_ (Figure 2).

Regarding the literature data, Zhao and Vanderbilt [61] obtained in their first-principles calculations that orientationally averaged static dielectric constants should be 29, 70, and 16 for the cubic, tetragonal, and monoclinic HfO_2_ phases, respectively. In this connection, the permittivity values measured in the present study might have been related to the polymorphic composition of the films under discussion (Figure 2). The permittivity values extending to 40 (Figure 4) could thus be due to the presence of the tetragonal HfO_2_ phase in the films. In this case, the value of the relative permittivity, although lower than that predicted by the first-principles calculations, could herewith be reasoned by the reduced tetragonality of the *t*′ form or possible co-existence of tetragonal, cubic, and amorphous phases in the corresponding films. In contrast, the relative permittivity value as high as 25 (Figure 4), measured for the undoped HfO_2_ film was higher than that attributed to the monoclinic HfO_2_ [61]. This might, supposedly, become explained by a minor contribution of metastable phases that could also be present in the films.

In earlier studies of Pr-doped HfO_2_ thin films that were grown by chemical solution deposition method and annealed at 800 °C, relative permittivity values of 23.5, 29.7, and 32.1 were obtained at Pr concentrations of 5, 10, and 15 mol%, respectively, while the corresponding value for an undoped HfO_2_ film was determined to be 18.3 [6]. The crystalline phases observed were the monoclinic one in the undoped films, the orthorhombic phase in the films with 5 mol% Pr, and the cubic phase in the films with 10 and 15 mol% Pr [6]. Comparing these results with those of theoretical calculations [61] and with the permittivity values obtained for ALD films in our work (Figure 4), one can conclude that the formation of the tetragonal structure was the main reason for the high permittivity values of our films deposited using a HfO_2_:PrO_x_ cycle ratio of 5:1.

The decrease in the permittivity observed with the decrease of the HfO_2_:PrO_x_ cycle ratio to 2:1 and increase of the Pr/(Pr + Hf) atomic ratio from 0.10 to 0.23 was probably caused by a decreasing amount of tetragonal phase in the films and/or decrease in the tetragonality of the *t*′ form of HfO_2_ with increasing concentration of Pr. The decrease in the tetragonality of the *t*′ form with increasing concentrations of rare-earth dopant in HfO_2_, has been observed, for instance, in the case of yttria-doped HfO_2_ [60]. Unfortunately, because of relatively wide reflections in the GIXRD patterns of our films (Figure 2), revealing this kind of change in the crystal structure was not possible in the present work.

Another effect that might have contributed to the permittivity decrease observed with the increase of the Pr/(Pr + Hf) atomic ratio from 0.10 to 0.23 was the doping anisotropy that was most significant in the films grown with a HfO_2_:PrO_x_ cycle ratio of 5:1. In the growth direction of these films, the Pr concentration varied with a period of 0.8–1.0 nm, approximately. This value was evidently larger than the in-plane distance between Pr^3+^ or Pr^4+^ ions, provided that the solid-phase diffusion did not lead to uniform distribution of Pr in the films. For comparison, the Pr concentration was expected to vary with a period of 0.5–0.6 nm in the growth direction of the films deposited using a HfO_2_:PrO_x_ cycle ratio of 2:1. In this case, the period of dopant concentration variation was closer to the in-plane distances between Pr^3+^ or Pr^4+^ ions and, for this reason, the Pr distribution was more uniform than that in the films deposited with a HfO_2_:PrO_x_ cycle ratio of 5:1.

Finally, the lower permittivity of PrO_x_ compared to that of tetragonal HfO_2_ could be a reason for the permittivity decrease observed for Pr-doped HfO_2_ with the decrease of the HfO_2_:PrO_x_ cycle ratio from 5:1 to 2:1. According to the literature data, the relative permittivity values of polycrystalline PrO_x_ films have ranged from 8 to 26 [62,63] being higher for Pr_2_O_3_ and lower for PrO_2_-rich films [62]. Therefore, to obtain the highest permittivity values, the Pr concentration in the Pr-doped HfO_2_ films should not exceed significantly the value corresponding to the transition from the monoclinic to the tetragonal phase.

The conductivity of metal oxide dielectrics is directly related to their bandgap widths. On the other hand, doping with foreign elements—otherwise beneficial in terms of the stabilization of high-permittivity phases—might introduce parasitic states in the bandgap which could, presumably, increase the conduction currents. For instance, in the case of doping HfO_2_ with praseodymium (oxide), Pr^3+^ ions have energy levels in the bandgap of HfO_2_ [64]. At the same time, however, the optical bandgap increased from 5.55–5.65 eV, determined for undoped HfO_2_, to 5.72–5.78 eV in the doped films, where Pr/(Pr + Hf) = 0.15–0.20 and a tetragonal phase was formed [36]. Notably, also the theoretical calculations have predicted a wider bandgap for the metastable tetragonal/cubic HfO_2_, compared to that of stable monoclinic HfO_2_ [65]. Therefore the bandgap widening compensates, at least to some extent, for the contribution of the additional states generated in the bandgap by doping, thereby enabling the formation of HfO_2_:PrO_x_ films appreciably insulating in the capacitor structures and in the low resistance state of RS devices.

### 3.3. Resistive Switching

**Figure 5:**
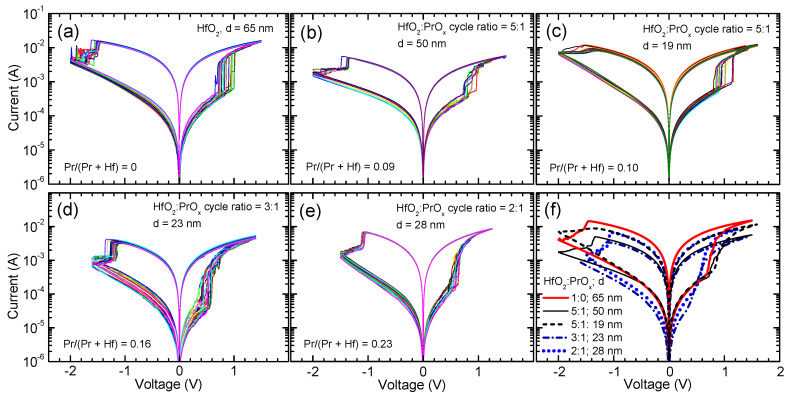
Current voltage envelope curves describing resistive switching of HfO_2_:PrO_x_ films as-deposited on Ru electrode substrates. HfO_2_:PrO_x_ deposition cycle ratios, Pr/(Pr + Hf) atomic ratios, and film thicknesses are indicated by labels in panels (**a**–**e**), where multiple (6–20) current–voltage curves are presented for the same film and device, measured consecutively and presented in different colors to enable better distinction. In panel (**f**), the averaged current–voltage curves are depicted comparatively.

The HfO_2_ and HfO_2_:PrO_x_ films, which were deposited in this work on Ru electrodes and supplied with Ti top electrodes, demonstrated RS behavior, expressed by clear current–voltage envelope curves characteristic of RRAM devices (Figure 5). The electroforming voltages did not vary systematically with the HfO_2_:PrO_x_ ratio. All the samples demonstrated, however, an abrupt forming transitions at 11–13 V to a current value of 1 μA set as a compliance limit in order to prevent irreversible breakdown.

The nature of the switching process (Figure 5) was bipolar, meaning that in the SET process, the conductive filaments were formed applying positive voltage in relation to the Ru electrode, while in the RESET process, the conductive paths were disrupted by applying voltage of the opposite polarity. The smallest electrodes with the average area of 0.002 mm^2^ provided the most reliable results, that is, the initial electroforming process did not typically cause the dielectric breakdown, which otherwise would indicate issues with lateral homogeneity in the switching media.

**Figure 6:**
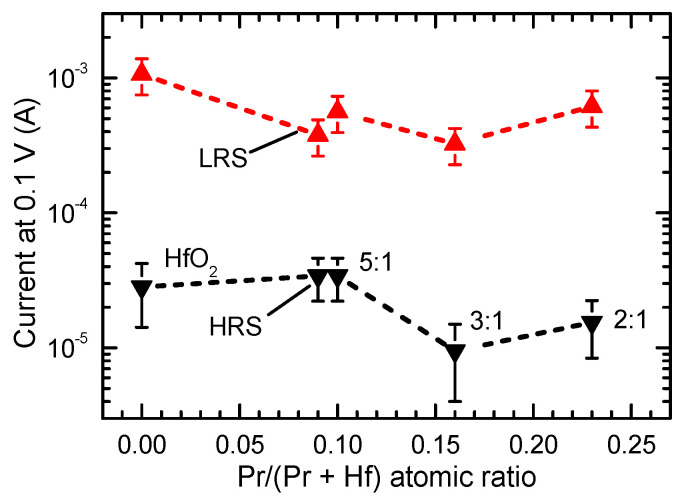
Current values recorded in low resistance state (LRS) and high resistance state (HRS) at a voltage value of 0.1 V (see Figure 5). The HfO_2_:PrO_x_ ALD cycle ratios are indicated by labels at corresponding data points.

The results depicted in Figure 5 and Figure 6 demonstrate that the Pr concentration in the RS layer influenced the commutation voltages, current values in the low-resistive state (I_LR_) and high-resistive state (I_HR_) as well as the I_LR_/I_HR_ ratio. The increase in the Pr/(Pr + Hf) atomic ratio from 0 to 0.10 caused a decrease in I_LR_ (Figure 5a–c and Figure 6) and a minor increase in the set voltages (Figure 5a–c). At the same time, no considerable changes in I_HR_ appeared (Figure 6). As a result, the I_LR_/I_HR_ ratios of the films that were grown using a HfO_2_:PrO_x_ cycle ratio of 5:1 were lower than that of un-doped HfO_2_.

Comparison of the data for films with similar Pr/(Pr + Hf) atomic ratios (0.09 and 0.10) and very different thicknesses (50 and 19 nm, respectively) revealed minor differences in I_LR_ (Figure 5b,c and Figure 6). However, as can be seen in Figure 6, these differences were comparable to the experimental uncertainty related mainly to the deviation of results in successive measurements of the current-voltage (I-V) curves.

With the increase of the Pr/(Pr + Hf) atomic ratio to 0.16, obtained at a HfO_2_:PrO_x_ cycle ratio of 3:1, the set and reset voltages and I_HR_ decreased while I_LR_/I_HR_ increased to a level exceeding that of undoped HfO_2_ (Figure 5d and Figure 6). The further increase of the Pr/(Pr + Hf) atomic ratio to 0.23, observed in the films grown with a HfO_2_:PrO_x_ cycle ratio of 2:1, did not influence the commutation voltages and I_LR_/I_HR_ significantly (Figure 5d–f). However, I_LR_ and I_HR_ somewhat increased when compared to the corresponding values of the film grown using a HfO_2_:PrO_x_ cycle ratio of 3:1 (Figure 6).

**Figure 7:**
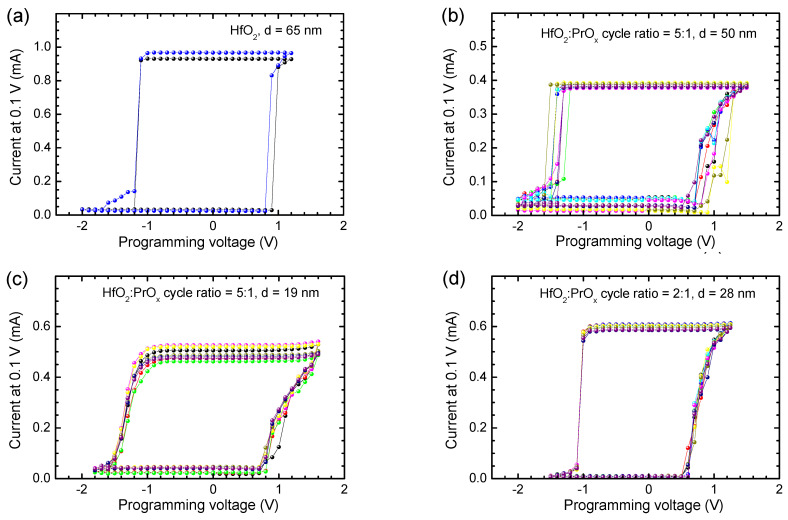
Current recorded at 0.1 V as a function of programming voltage used for biasing of the (**a**) HfO_2_ and (**b**–**d**) HfO_2_:PrO_x_ media before the current measurement. The HfO_2_:PrO_x_ cycle ratios used for growing the films and film thicknesses are indicated by labels. Different colors correspond to different memory maps recorded for the same film and device. The Pr/(Pr + Hf) atomic ratios were (**b**) 0.09, (**c**) 0.10, and (**d**) 0.23.

Besides the envelope I-V curves describing current values measured at the variable bias voltage values (Figure 5), memory maps were recorded at a constant reading voltage as well (Figure 7). In the latter case, the conduction currents were read at a voltage of 0.1 V in between the sequential programming voltage pulses. Two clearly defined plateaus were reached between sequential SET and RESET events (Figure 7). I-V loops, which can be termed as memory maps, were thus formed with prominently expressed memory windows between the high and low current states during multiple voltage loops (Figure 7).

The memory maps supported the results depicted in Figure 5 and Figure 6, showing that narrower windows between the low and high resistivity states, that is lower I_LR_/I_HR_ ratios, were obtained for the reference HfO_2_ films (I_LR_/I_HR_ ≈ 30, Figure 7a) and especially for the films deposited using HfO_2_:PrO_x_ cycle ratio of 5:1 (I_LR_/I_HR_ ≈ 10, Figure 7b,c). The window between the low and high resistivity states was much wider in the case of samples containing oxide media grown with HfO_2_:PrO_x_ cycle ratios of 3:1 (not shown) and 2:1 (I_LR_/I_HR_ ≈ 60, Figure 7d). Hence, rather than being a monotonic function of the Pr-content, the I_LR_/I_HR_ ratio possessed a minimum value at Pr/(Pr + Hf) atomic ratios of 0.09–0.10, that is, at the HfO_2_:PrO_x_ cycle ratio of 5:1. Earlier a significant dependence of RS performance on the doping level has been reported for Pr-doped ZnO films where the best functional ratio between resistance states appeared in the case of a certain stoichiometry [66]. The argument of an optimal composition has also been supported by the first-principles calculations made on HfO_x_-based films [67]. Furthermore, thicknesses may also become more optimized at certain film stoichiometries [68], and would, consequently, require further more detailed parametrization.

Comparing the results depicted in Figure 5, Figure 6 and Figure 7 with the GIXRD patterns (Figure 2), one can see that the I_LR_ decrease, caused by the increase of Pr/(Pr + Hf) to 0.09–0.10, was evidently related to the formation of the metastable phase in the films. The wider bandgap of this phase [36] and possible compensation of oxygen vacancies because of Pr-doping are the plausible reasons for this I_LR_ decrease. A probable reason for why the I_HR_ value did not decrease and, correspondingly, the I_LR_/I_HR_ value decreased was the presence of some inclusions of monoclinic phase in the films deposited using a HfO_2_:PrO_x_ cycle ratio of 5:1. An evidence of the monoclinic phase is a tail on the right side of the 30.3° reflection in the diffraction pattern of the 50-nm thick films grown with this cycle ratio (Figure 2). This tail was evidently related to the contribution of the 111 reflection of the monoclinic phase. Although the small amounts of the monoclinic phase were not able to have a marked effect on the low-resistive state, the contribution of those to the much lower I_HR_ values was still an expected result. Correspondingly, the absence of the monoclinic phase in the films grown with HfO_2_:PrO_x_ cycle ratios of 3:1 and 2:1 and the formation of a more homogeneous crystalline phase in these films were reasons for lower I_HR_ and higher I_LR_/I_HR_ values obtained. The latter films also possessed somewhat lower commutation voltages (Figure 5f and Figure 7) than the rest of the samples did. This observation implies that the media which contain higher amounts of praseodymium and only metastable nanocrystalline phases might become better suited to low-power applications. Higher mobility of oxygen ions in these films might be one possible reason for lower commutation voltages observed.

Endurance measurements were carried out for samples with different doping levels to determine their reliability and resistance state variability during fast writing, erasing, and reading operations. In these measurements, a current compliance of 5 mA was set in order to prevent the irreversibly breaking of dielectrics when applying the commutation voltages in a short period of time. The use of compliance has been shown to improve the endurance characteristics in HfO_x_-based RRAM [69]. However, the same functional windows between the high and low resistance states were not expected when comparing the results of endurance studies with the current-voltage envelope curves or memory maps.

**Figure 8:**
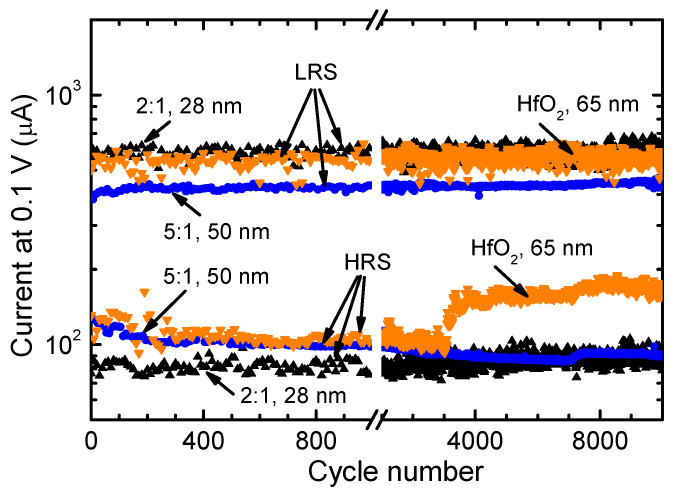
Endurance characteristics of HfO_2_:PrO_x_ switching media measured in the small-signal regime. The HfO_2_:PrO_x_ cycle ratios used for ALD of dielectrics and the dielectric layer thicknesses are indicated by labels at corresponding curves. I_LR_ and I_HR_ values for every tenth measurement cycle are depicted in the figure.

First of all, the endurance characteristics presented in Figure 8 revealed higher stability of Pr-doped HfO_2_ media compared to that of undoped HfO_2_. In the case of samples with undoped HfO_2_, the first instabilities in I_LR_ as well as I_HR_ appeared after applying a few hundred cycles, while a marked increase in I_HR_ was observed after 3000 cycles. In contrast, the RS layer grown using a HfO_2_:PrO_x_ cycle ratio of 2:1 showed very stable I_LR_ as well as I_HR_ values up to the end of the endurance measurements (Figure 8). Therefore, the Pr-doping of HfO_2_ and/or formation of metastable (tetragonal) phase in the RS medium play a significant in producing memory cells with superior RS performance.

An even more interesting performance was observed in the case of samples with Pr-doped HfO_2_ deposited using a HfO_2_:PrO_x_ cycle ratio of 5:1 that yielded films with Pr/(Pr + Hf) atomic ratios of 0.09–0.10. Figure 8 demonstrates that with an increasing number of switching cycles, the I_HR_ value of a 50-nanometer thick film considerably decreased while the I_LR_ value increased resulting in a marked increase in the I_LR_/I_HR_ ratio. It is noteworthy that a similar performance was observed for a 19-nanometer thick films with a Pr/(Pr + Hf) atomic ratio of 0.10. In both cases, the changes in I_HR_, I_LR_, and I_LR_/I_HR_ were relatively smooth without significant random fluctuations of the current values. Therefore, it seems that the conductive filaments were additionally electroformed (stabilized) in this kind of RS medium during the endurance studies. Phase transitions leading to the formation of a more uniform crystal structure and/or more uniform distribution of Pr^3+^/Pr^4+^ ions in the vicinity of switching filaments can be considered as the main mechanisms causing this kind of modification of the RS medium.

Comparison of our results with literature data confirmed the excellent performance of Pr-doped HfO_2_ studied in this work compared to that of many other HfO_2_-based undoped and doped RS media. Typical I_LR_/I_HR_ values reported [16,70,71,72,73,74,75] have ranged from one to two orders of magnitude being well comparable to the corresponding values obtained in our work. However, I_LR_/I_HR_ values as high as three orders of magnitude have also been reported for samples with optimized electrode structure [76]. In most cases, the endurance tests, if performed, have been limited to 1000 switching cycles [70,71,73,75] but reports about endurance tests up to 10^5^ [72,74] and 10^8^ [16] switching cycles have also been published. Comparing the results described in the latter papers with those obtained in our experiments one can still conclude that after optimization of the device processing procedures, Pr-doped HfO_2_ can also become a promising candidate for application in resistive switching random access memories.

## 4. Conclusions

Our studies of Pr-doped HfO_2_ thin films grown by ALD on ruthenium electrodes revealed superior properties of these films for different electronic applications. The films grown using HfO_2_:PrO_x_ ALD cycle ratios, ranging from 5:1 to 2:1, crystallized in the tetragonal phase while the Pr/(Pr + Hf) atomic ratio ranged from 0.09 to 0.23 in these films. The relative permittivity values as high as 37–40 were measured for the films with Pr/(Pr + Hf) atomic ratios of 0.09–0.10. No considerable dependence of the permittivity on the measurement frequency was observed in a frequency range of 0.01–1 MHz. As the films with this composition have also high bandgap values (5.72–5.78 eV), as revealed in our earlier work, they could successfully be applied as capacitor and gate dielectrics.

Additionally, stable-resistive switching performance was obtained in metal–dielectric–metal structures with HfO_2_:PrO_x_ dielectrics, ruthenium bottom electrodes, and titanium top electrodes. In the structures with as-grown dielectrics, the I_LR_/I_HR_ ratios ranged from 10 in the case of dielectrics with Pr/(Pr + Hf) atomic ratios of 0.09–0.10 to 60 in the case of dielectrics with Pr/(Pr + Hf) atomic ratios of 0.16–0.23. The resistive switching layers with Pr/(Pr + Hf) atomic ratios of 0.16–0.23 showed also lower I_LR_, I_HR_, and commutation voltage values compared to corresponding parameters recorded for devices with undoped HfO_2_ dielectric. The endurance measurements demonstrated excellent stability of resistive switching in Pr-doped HfO_2_ during 10^4^ switching cycles. Thus, Pr-doped HfO_2_ could be considered as a promising material for application in resistive switching memory devices as well.

## Data Availability

Not applicable.
